# Prevalence of Elevated D-dimer Levels in Confirmed COVID-19 Cases in Intensive Care Unit of a Tertiary Care Centre of Western Nepal

**DOI:** 10.31729/jnma.6284

**Published:** 2021-03-31

**Authors:** Prabin Khatri, Krishna Kumar Agrawal, Dipesh Sharma, Pradip Chhetri, Aryan Neupane, Rano Mal Piriyani, Pawan Puspa Baral, Suman Raj Sapkota, Ashish Banjade, Ashmita Chhetri, Subarna Bhandari, Swaraj Bharali

**Affiliations:** 1 Department of Internal Medicine, Universal College of Medical Sciences, Bhairahawa, Nepal; 2 Karuna Hospital, Kathmandu; 3 Department of Community Medicine, Universal College of Medical Sciences, Bhairahawa, Nepal; 4 Universal College of Medical Sciences, Bhairahawa, Nepal; 5 Department of Anesthesiology, Universal College of Medical Sciences, Bhairahawa, Nepal

**Keywords:** *COVID-19*, *d-dimer*, *intensive care unit*

## Abstract

**Introduction:**

D-dimer is currently the best available marker for COVID-19 associated hemostatic abnormalities. This study aims to find out the prevelance of elevated D-dimer levels in confirmed COVID-19 cases in intensive care unit of a tertiary care hospital of western Nepal.

**Methods:**

This descriptive cross-sectional study was conducted among 95 patients admitted to COVID Intensive Care Unit of a teriary care centre from August 2020 to January 2021 after taking ethical clearence from Institutional Review Committee in order to determine the D-dimer levels in confirmed COVID-19 cases. D-dimer value was measured at the admission and the highest D-dimer value was recorded during the course of hospital stay with the risk of mortality in confirmed COVID-19 cases. The normal range of D-dimer was taken as <0.35 mg/dl as per our hospital laboratory standards. Convenience sampling method was used. Data entry and descriptive analysis were done in Statistical Package for the Social Sciences version 25.0, point estimate at 95% Confidence Interval was calculated along with frequency and proportion for binary data.

**Results:**

Out of total 95 cases of COVID-19 included in this study, 25 (89.3%) patients with age ≥65 years and 42 (62.69%) patients aged <65 years had elevated D-dimer on admission. Data showed that 29 (67.4%) patients having elevated D-dimer at admission had mortality.

**Conclusions:**

Elevated D-dimer levels was frequently seen in patients admitted in Intensive Care Unit with COVID-19. Our study suggested that measurement of D-dimer may guide in clinical decision making.

## INTRODUCTION

COVID-19 triggers coagulation cascade, which is linked with a hypercoagulable state and worse clinical outcomes.^1,2^ Presence of thrombi within the pulmonary vasculature is associated with diffuse alveolar damage seen in most patients who died from COVID-19.^2,3^ Hemostasis based laboratory monitoring and treatment seems crucial owing to dysfunctional coagulation in COVID-19.^4^ D-dimer is currently the best available marker for COVID-19 associated hemostatic abnormalities.^5^

The determination of circulating D-dimer concentrations is a sensitive test in clinical practice to diagnose thrombotic states, including pulmonary embolism and Disseminated Intravascular Coagulation (DIC).^6^ Increased D-dimer values have often been found in COVID-19 patients and were higher in the cases who are critically ill or those who die.^7-13^

The aimof our study was to find out the prevelance of elevated D-dimer levels in confirmed COVID-19 cases in intensive care unit of a tertiary care hospital of western Nepal.

## METHODS

We conducted a descriptive cross-sectional study in the patients admitted to COVID-19 Intensive Care Unit (ICU) of Universal College of Medical Sciences (UCMS) in order to determine the D-dimer levels in confirmed COVID-19 cases. Patients from August 2020 to January 2021 were enrolled after taking ethical clearence from Institutional Review Committee at Universal College of Medical Sciences (Ref No: 155/20). Convenience sampling was done and sample size was calculated using formula:

$$\begin{array}{ll} \text{n} & ={\text{Z}}^{\text{2}}\times \text{p}\times \text{q}/{\text{e}}^{\text{2}}\\ & ={\left(1.96\right)}^{2}\times (0.5)\times (1-0.5)/{\left(0.11\right)}^{\text{2}}\\ & =30.41 \end{array}$$

Where,

n = required sample size,
Z = 1.64 at 90% Confidence Interval
p = prevelance of elevated D-dimer levels in confirmed COVID-19 cases, 50%
q = 1-p
e = margin of error, 11%

Thus, the calculated sample size was 79. During that period a total of 138 patients were admitted in the COVID-ICU out of which 95 fulfilled the study inclusion criteria. Hence, samples from 95 patients were included. The patient aged more than 16 years with positive real-time polymerase chain reaction (RT-PCR) assay for SARS-CoV-2 and requiring ICU management were included after obtaining a written consent.

The criteria for ICU management of confirmed COVID-19 cases as per Nepalese Society of Critical Care Medicine were: ^14^

1. Respiratory rate > 24/min,
2. SPO2 <94 on room air at rest,
3. Confusion/drowsiness,
4. Systolic BP <90 mmHg or diastolic BP <60mmHg and
5. Those with high risk for severe disease
  - Age >60 years without any comorbidities
  - Cardiovascular risk including hypertension
  - Diabetes Mellitus other immune compromised states
  - Chronic lung/liver/kidney disease.

Pregnancy, cancer, hematologic malignancy, chronic liver disease, deep vein thrombosis, pulmonary embolism, surgery or trauma within 30 days, patients without D-dimer testing upon admission and those who do not give written consent were excluded from the study.

GPP-100 Specific Protein Analyzer for quantitative determination of D-dimer in human plasma was used for the D-dimer testing. The principle applied is Immunonephelometry. This method measures the light scattered by the insoluble immune complexes formed between D-dimer in the sample and its specific antibodies, and the amount of light scattered is directly proportional to the concentration of D-dimer when the antiserum is in excess. Reference range (less than 0.35 mg/L) was considered as normal D-dimer and D-dimer greater than 0.35 mg/L was considered as elevated D-dimer according to UCMS laboratory range. D-dimer values were collected at two time points: at the admission, and highest value during hospital stay.

Data were entered and analyzed in Statistical Package for the Social Sciences version 25.0 (SPSS). Descriptive statistics like frequency, percentage, mean and standard deviation were obtained.

## RESULTS

Out of total 95 cases of COVID-19 included in this study, 25 (89.3%) patients with age ≥65 years and 42 (62.69%) patients aged <65 years had elevated D-dimer on admission. Out of total 59 (62.1%) male, Fourty four (74.6%) male and out of total 36 (31.9%) female, 23 (63%) female had elevated D-Dimer.

The mean age was 56.2±14.9 ranging from 16 years to 90 years. Shortness of breath 78 (82.1%), fever 67 (70.5%), and cough 60 (63.1%) were among the leading presenting complains in our study population. In addition, 12 (12.6%) had anosmia, 16 (16.8%) had diarrhea, and 14 (14.7%) had headache. Among the comorbidities, Hypertension was present in 32 (33.7%), Diabetes mellitus was present in 23 (24.2%), Chronic Obstructive Pulmonary Disease (COPD) in 3 (3.1%), Chronic Kidney Disease (CKD) in 3 (3.1%), and Bronchial Asthma was seen in 1 (1.01%). Out of the total 95 patients, mortality was observed in 43 (45.3%). The mean number of days of ICU stay was 8.2±4.2 days (Table 1).

**Table 1:** Demographic and clinical outcomes of COVID-19 patients.

Variables		n (%)
Gender	Male	59 (62.1)
	Female	36 (31.9)
	16-64	67 (70.5)
Age (Mean±SD)	≥65	28 (29.5)
	56.2 ± 14.9 years	
	Fever	67 (70.5)
	Shortness of Breath	78 (82.1)
Presenting Complains	Myalgia	26 (27.4)
	Cough	60 (63.1)
	Anosmia	12 (12.6)
	Headache	14 (14.7)
	Diarrhoea	16 (16.8)
	Diabetes Mellitus	23 (24.2)
	Hypertension	32 (33.7)
Comorbidities	COPD	3 (3.1)
	Chronic Kidney Disease	3 (3.1)
	Bronchial Asthma	1 (1.01)
Outcome	Survival	52 (54.7)
	Mortality	43 (45.3)
Number of days in ICU	8.2±4.2 days	

Similarly, 47 (78.3%) patients with cough and 61 (78.2%) of patients with shortness of breath had elevated D-dimer on admission. We observed that most of the patients having comorbidities had elevated D-dimer. The results showed that 100% patients with CKD, bronchial asthma had elevated D-Dimer. Similarly Patients having Diabetes Mellitus, about 81.8% patients had elevated D-Dimer. Our data showed that 29 (67.4%) patients having elevated D-dimer at admission had mortality (Table 2).

**Table 2:** Distribution of variables among normal and elevated D-Dimer.

Variable		Normal D-dimer n (%)	Elevated D-dimer n (%)
Gender	Male	15 (25.4)	44 (74.6)
	Female	13 (36.1)	23 (63.9)
Age	16-64 years	25 (37.31)	42 (62.69)
	≥65	3 (10.7)	25 (89.3)
	Fever	22 (32.8)	45 (67.2)
	Shortness of Breath	17 (21.8)	61 (78.2)
	Myalgia	9 (34.6)	17 (65.4)
Presenting Complains	Cough	13 (21.7)	47 (78.3)
	Anosmia	6 (50)	6 (50)
	Headache	5 (35.7%)	9 (64.3)
	Diarrhoea	6 (37.5)	10 (62.5)
	Diabetes Mellitus	6 (18.2)	27 (81.8)
Comorbidities	Hypertension	12 (37.5)	20 (62.5)
	COPD	1 (33.3)	2 (66.7)
	CKD	0 (0.0)	3 (100)
	Bronchial Asthma	0 (0.0)	1 (100)
Outcome	Mortality	14 (32.6)	29 (67.4)

The results showed that 40 (88.1%) male & 20 (86.1%) female had highest value of elevated D-Dimer. Twenty eight (100%) patients with age ≥65 years and 55 (82.09%) patients with age less than 65 years had elevated D-dimer during their hospital stay. Similarly out of different presenting complains, those who presented with shortness of breath had elevated levels of D-dimer during their hospital stay. The results showed that 31 (93.3%) patients with diabetes developed elevated D-dimer levels during their hospital stay. All the 43 patients who died had elevated D-dimer levels during their hospital stay. This results showed that patients having elevated D-dimer had high risk of mortality (Table 3).

**Table 3:** Variables distributed among Normal and Elevated D-Dimer at highest level.

Variable		Highest value of D-dimer within Normal Range n (%)	Highest value of D-dimer Elevated n (%)
Gender	Male	7 (11.9)	52 (88.1)
	Female	5 (13.9)	31 (86.1)
Age	16-65 years	12 (17.91)	55 (82.09)
	≥65 years	0 (0.0)	28 (100.0)
	Fever	9 (13.4)	58 (86.6)
	Shortness of breath	6 (7.7)	72 (92.3)
	Myalgia	1 (3.8)	25 (96.2)
Presenting Complaint	Cough	5 (8.3)	55 (91.7)
	Anosmia	0 (0.0)	12 (100)
	Headache	1 (7.1)	13 (92.9)
	Diarrhoea	3 (18.75)	13 (81.25)
	Diabetes Mellitus	2 (6.1)	31 (93.3)
	Hypertension	5 (15.6)	27 (84.4)
Comorbidities	COPD	0 (0)	3 (100)
	Chronic Kidney Diseases	0 (0)	3 (100)
	Bronchial Asthma	0 (0)	1(100)
Outcome	Mortality	0 (0)	43 (100)

Our results showed that out of 83 patients with elevated D-dimer levels, 14 (16.87%) had D-dimer values between 0.35-0.99 mg/L, 23 (27.71%) had D-dimer values between 1-2 mg/L and 46 (55.42%) had D-dimer values >2 mg/L. Among patients with different levels of elevated D-dimer, no patient with D-dimer values 0.35-0.99 mg/L died, 11 (47.82%) patients with D-dimer levels between1-2mg/L died, and 32 (69.56%) patients with D-dimer levels >2 mg/L died (Table 4).

**Table 4:** Mortality among different levels of elevated D-dimer.

D-dimer levels	Number of Patients n (%)	Mortality among different levels of elevated D-dimer n (%)
0.35- 0.99 mg/L	14 (16.87)	0 (0)
1-2 mg/L	23 (27.71)	11 (47.82)
>2mg/L	46 (55.42)	32 (69.56)
**Total**	**83 (100)**	**43 (100)**

## DISCUSSION

In our study, we have identified elevated D-dimer level in significant number of the cases of RT-PCR positive COVID-19 patients admitted to COVID ICU. The D-dimer is a relatively small protein fragment that is present in the blood following degradation of blood clots by fibrinolysis. The determination of circulating D-dimer concentrations is a sensitive test in clinical practice to diagnose thrombotic states, including pulmonary embolism and DIC.^6^ Christensen et.al reported that increased D-dimer values has often been found in COVID-19 patients.^7^ This was also shown in our study where 87.36% of the total cases had elevated D-dimer levels at any time during their hospital stay.

In our study, elevated D-dimer levels was found to be higher in age group >65 years, 28 (100.0%) out of which 25 patients had elevated levels during admission. Also, 29 (67.4%) patients having elevated D-dimer at admission had mortality. All the 43 patients who died had elevated D-dimer levels during their hospital stay. These findings were comparable with study done by Mamta etal.^15^

In patients with confirmed COVID-19, D-dimer has been reported to be higher in cases who are critically ill or those who die.^7-13^ This finding is comparable to our study where all the cases with mortality had elevated D-dimer levels. In a study conducted by Zhang et al. there were 13 deaths during hospital stay out of which 12 were among patients whose D-dimer levels more than 2.0 mg/L.^16^ In our study 46 patients had D-dimer levels >2 mg/L out of which 32 showed mortality.

Mishra et al. demonstrated COVID-19 patient with diabetes had higher D-dimer levels which is corresponding to our study where 27 (81.8%) patients with diabetes had elevated D-dimer levels.^17^ Since diabetes is a prothrombotic state patients with diabetes have increased risk of thromboembolic events due to imbalance between clotting factor and firbrinolysis.^18^

This study cannot be generalized as it is a single centered study and only in hospital mortality were included in the study. Further assessment of the discharged patients and their D-dimer levels were not done. A larger sample and multicenter study are needed to address these issues.

## CONCLUSIONS

Elevated D-dimer levels was frequently seen in patients admitted in Intensive Care Unit with COVID-19. In patients with confirmed COVID-19, coagulopathy was found to be a major complication and was closely related to the disease severity and mortality. All patients who died had elevated D-dimer levels anytime during their hospital stay. Our study suggested that measurement of D-dimer can guide in clinical decision making.
